# Safety and efficacy of extended versus standard interval dosing of natalizumab in multiple sclerosis patients: a systematic review and meta-analysis

**DOI:** 10.1007/s13760-024-02480-6

**Published:** 2024-03-08

**Authors:** Eslam Mohammed Rabea, Mohamed Mohamed Belal, Abdelrahman H. Hafez, Ashraf Hassan Elbanna, Mahmoud Ahmed Khalifa, Anas Zakarya Nourelden, Nada H. Mahmoud, Mohamed Sayed Zaazouee

**Affiliations:** 1 Faculty of Medicine, Alexandria University, Alexandria, Egypt; 2 Faculty of Medicine, Al-Azhar University, Cairo, Egypt; 3 Faculty of Medicine, Al-Azhar University, Assiut, Egypt

**Keywords:** Natalizumab, Extended interval dosing, EID, Standard interval dosing, SID, Multiple sclerosis, Meta-analysis

## Abstract

**Background:**

Multiple sclerosis (MS) is a chronic inflammatory, immune-mediated disease affecting the central nervous system. Natalizumab, an FDA-approved monoclonal antibody for MS, has been explored for its off-label extended interval dosing (EID), suggesting a potential reduction in the risk of progressive multifocal leukoencephalopathy (PML) compared to standard interval dosing (SID). Our objective was to assess the efficacy and safety of EID in comparison to SID for natalizumab treatment in patients with MS.

**Methods:**

We searched PubMed, Embase, WOS, Scopus, Ovid, Science Direct, Clinical trials.gov, and Cochrane Library. Our assessed outcomes were clinical relapses, MRI activity, change in expanded disability status scale [EDSS], and the risk of PML. The EID group was defined as 5 to 8 weeks [EID (Q5-8W)]. The analysis was conducted using RevMan ver. 5.4. The effect estimates were presented as a risk ratio [RR] or mean difference with 95% confidence intervals [CI] using SID group as the reference for comparisons.

**Results:**

Fourteen studies met our inclusion criteria: 2 RCTs, 1 switched single-arm trial, and 12 observational studies. No significant differences were found in all efficacy outcomes of interest. Risk of clinical relapses [RR = 0.90, (95%CI 0.80, 1.02)], risk of new or newly enlarging T2 hyperintense MRI lesions [RR = 0.78, (95%CI 0.59, 1.04)], risk gadolinium enhancing lesions [RR = 1.30, (95%CI 0.98, 1.72)], change in EDSS [MD = 0.09 (95%CI − 0.57, 0.76)], risk of PML [RR = 1.09, 95%CI (0.24, 4.94)].

**Conclusion:**

In summary, our meta-analysis indicates that natalizumab maintains its effectiveness under extended interval dosing [up to 8 weeks], presenting comparable risks for clinical relapses, MRI lesions, EDSS, and PML. Caution is advised given study limitations and heterogeneity. Robust conclusions necessitate well-designed high-quality prospective studies.

**Supplementary Information:**

The online version contains supplementary material available at 10.1007/s13760-024-02480-6.

## Introduction

Multiple sclerosis [MS] is a chronic inflammatory neurological immune-mediated disease of the central nervous system [CNS] arising from the interaction of genetic and environmental factors. It is characterized by inflammatory demyelination of the white and grey matter in CNS mediated by the complex interaction and dysregulation of multiple immune cells that lead to chronic inflammation, demyelination, and subsequent neurodegeneration [1, 2]. The global prevalence of MS rose from 2.3 million in 2013 to 2.8 million in 2020, and it reached 2.9 million in 2023 [3].

Natalizumab is a humanized monoclonal antibody against α4β1 integrin. It blocks their binding to the endothelial receptors, thus reducing the entrance of lymphocytes to the CNS through the blood–brain barrier [4–6]. This was associated with decreased inflammation and improved clinical and radiological activity [7]. Natalizumab was approved to be used intravenously with a fixed dose [300 mg] every four weeks [Q4W] [6, 8]. Several studies demonstrated its efficacy in treating MS [9–11]. However, it was associated with an increased risk of developing progressive multifocal leukoencephalopathy [PML], a rare opportunistic infection caused by the reactivation of the latent John Cunningham virus [JCV] [12, 13]. It was found to be more associated with patients positive for anti-JCV serology, prior immunosuppressive intake, or receiving infusions for more than two years [13].

Van Kempen et al. found that the natalizumab concentration remained high at the time of re-dosing in most participants [14]. Stopping natalizumab for ≥ 3 months after 1–2 years of the standard interval regimen [SID] has been suggested to reduce PML incidence. Still, it was associated with a number of MS relapses [15]. Grimaldi et al. found that the risk of MRI activity rose by 1.34-fold per each week of delay from the SID [16]. However, another study revealed that extended interval dosing [EID] [Q > 4W] of natalizumab was associated with lower PML risk in MS patients who tested positive for anti-JCV antibody [17]. These controversies between safety and efficacy have led to more research efforts to test the efficacy of natalizumab at various longer dosing intervals to decide on the safer regimen possible.

In the last decade, many studies have focused on this point. Some studies focused on the pharmacokinetics and pharmacodynamics point of view [18–20]. Other observational studies have focused on the differences in clinical effectiveness with doubtful results, which is the main focus of our meta-analysis [4, 21–23]. To our knowledge, there is no previous systematic review or meta-analysis on that subject. Therefore, our study aims to pool the current evidence of the efficacy and safety of EID of natalizumab compared to SID in MS patients.

## Methods

This systematic review and meta-analysis was performed in accordance with the PRISMA and Cochrane handbook guidelines [24, 25].

### Databases and search terms

Without any restrictions in dates or language, we searched PubMed, Scopus, WOS, Embase, Ovid, Science Direct, Clinical trials.gov, and Cochrane Library till June. 2023. We used these search terms; natalizumab, Tysabri, antegren, extended interval, EID, 6 weeks, multiple sclerosis, MS, RRMS, and disseminated sclerosis. This search was supported by an extensive manual search throughout the study period to check for any missed studies.

### Eligibility criteria

Randomized controlled studies [RCTs] and observational studies published in English were eligible for inclusion if comparing the EID [the intervention] of natalizumab with SID [the control] in MS patients [the population]. We included studies investigating the efficacy or safety of the EID strategy compared to the SID strategy [the outcomes]. Our study's standard dose of interest is 300 mg given by intravenous infusion. No limits were put for a certain age group, a certain definition of EID strategy, or a certain follow-up duration. We excluded reviews, editorials, case reports, case series, studies in a non-English language, and studies investigating different outcomes.

### Study selection and data extraction

The studies were exported to Rayyan to screen their titles and abstracts [26]. Two independent reviewers assessed each study. In case of any disagreement, a consensus with a third reviewer was made to solve the conflict. Two reviewers obtained and screened the full texts of potentially eligible studies. The final included studies were read carefully to extract the relevant data into Microsoft Excel spreadsheets. The summary and baseline characteristics of the enrolled patients in the included studies were extracted and tabulated. Sample sizes, countries, year of recruitment, study design, and assessed outcomes were extracted in the summary table. Mean age, male/female ratio, interval durations, treatment durations, follow-up durations, JCV[ +] patients, and prior use of other disease-modifying agents were extracted into the baseline table.

### Outcomes, analysis, and quality assessment

Outcomes of interest in this analysis were clinical relapses, MRI activity, PML, and change in the expanded disability status scale [Delta EDSS]. MRI new activity was represented in 2 outcomes; new or newly enlarging T2 hyperintense lesions and Gadolinium-enhancing lesions. If the study reported on different follow-up periods, we would consider the outcomes of the longer period. In Ryerson et al. [17], we extracted numbers of PML cases after 2 years only to make all follow-up periods as close as possible.

The statistical meta-analysis was conducted using Review Manager software ver.5.4. We used *I*^2^ statistics to describe the variation across the studies. An *I*^2^ > 50% or *P* < 0.1 indicates significant heterogeneity [27, 28]. A random-effects model was used in all analyses. In case of heterogeneity, a leave-one-out test was considered. Due to the observed variability among the studies in terms of the definition of EID, a subgroup analysis was done based on the extended interval durations. The effect estimates were shown as a risk ratio [RR] or mean difference with 95% CI. A funnel plot was generated with Review Manager software ver.5.4 to address publication bias.

The GRADE [Grades of Recommendation, Assessment, Development, and Evaluation] approach was used to assess the quality and strength of the evidence [29]. Each study was assessed for quality by two independent reviewers using the Cochrane Collaboration’s tool for assessing the risk of bias [25], the National Institutes of Health [NIH] quality assessment tool [30], and the Newcastle–Ottawa Scale [NOS] [31] according to their study designs.

## Results

### Search results

**Fig. 1:**
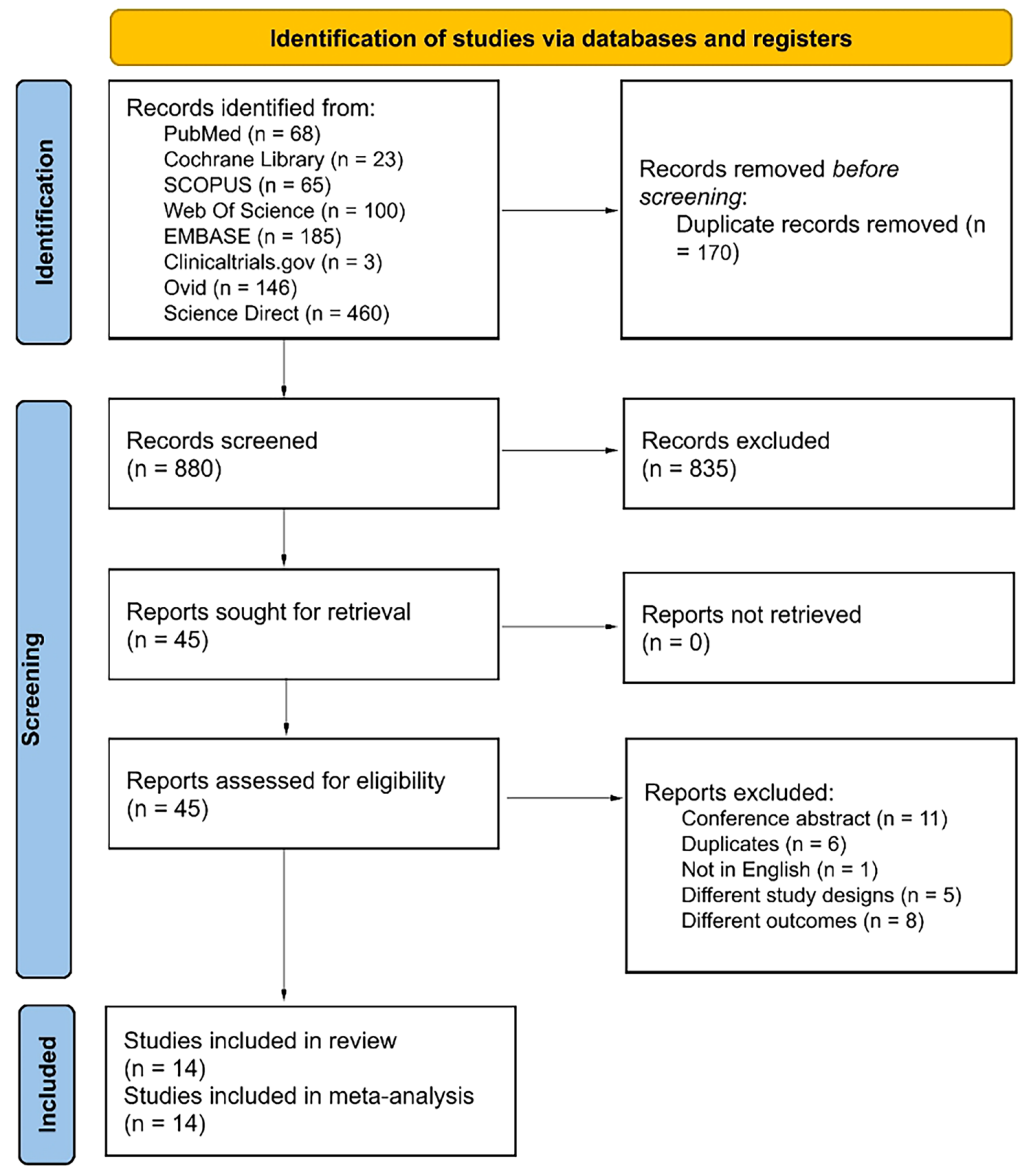
The PRISMA flow diagram

Our search resulted in a total of 880 references after removing the duplicates. 45 records were found relevant by title and abstract screening. After the full-text screening, 14 out of 45 records met our inclusion criteria [4, 17, 21–23, 32–40]. The PRISMA flowchart is shown in Fig. 1*.*

### Study characteristics

A total of 14 studies were found eligible. Two of them were RCTs [21], one was a switched single-arm trial [38], and the other 11 studies were observational. Data of the patients were retrieved from different databases: TOUCH database, Biogen’s Tysabri Global Safety Database, Tysabri Observational Program and many centers around the world, in the Americas, Belgium, Germany, Spain, France, Lebanon, and Iran. Clerico et al. [41] and De Mercanti et al. recruited their data from the same registry; that is why we included the data of the recent study—De Mercanti et al.—to avoid any overlapping outcomes [35].

Two third of patients were females, with the mean age being in their forties. The two arms of each study were patients who were stable on SID and switched to EID vs. those who remained on the SID. Studies have wide variability in the definition of EID. With the exception of Trojano et al. [21], in which the EID was defined as 12 weeks, all other studies defined the EID in a range from five weeks to eight weeks. SID differed slightly in its definition in the included studies ranging from four to five weeks. Also, there was variability in the treatment durations; however, the follow-up durations ranged from 12 to 24 months in most studies. The summary and baseline characteristics of enrolled patients in the included studies are shown in Tables 1, 2*.*

**Table 1:** Summary of the included studies

ID	Centers	Sample size, (EID/SID), *n*	Year of recruitment	Study design	Groups	Outcomes
Bomprezzi [33]	Vanderbilt University Medical Center and Barrow	96/361	September 2006 to April 2013	Retrospective cohort	Two different groups	Relapses and MRI activity
Butzkueven [23]	Tysabri Observational Program	219/219	2014 to November 2019	Retrospective cohort	Two different groups	ARR, and confirmed disability worsening
Chisari [34]	Italian MS Register	838/1254	1 June 2012 to 15 May 2018	Retrospective multicenter	Two different groups	Relapses, EDSS, ARR, NEDA-2, Progression index, and Confirmed disability improvement
De Mercanti [35]	14 Italian MS centers	129/187	March 2007 to March 2018	Retrospective multicenter	Two different groups	MRI activity
Foley [4]	89 multiple sclerosis centers across11 countries in the Americas, Europe, and Western Pacific	247/242	–	Randomized, controlled, open-label, phase 3b trial (NOVA)	Two different groups	Relapses, ARR, and MRI activity
Jeantin [39]	Monocentric, (Observatoire Français de la Sclérose en plaques, OFSEP) database	57/57	2020	Retrospective, self-controlled	Pre vs post switch	Relapses, MRI activity
Pelle [40]	5 different Frensh centers; Caen, Nice, Bobigny and Toulouse hospitals as well as Percy Military Hospital	147/156	2020	Retrospective multicenter	Two different groups	Relapses, EDSS, ARR, MRI activity, anti-JCV index
Riancho [36]	Sierrallana, in Cantabria, Spain	39/39	–	Retrospective cohort	Pre vs post switch	ARR, radiological activity, disability progression, and NEDA-3
Ryerson [37]	9 US MS centers	894/1080	2004	Retrospective cohort	Two different groups	Relapses, MRI activity, and ARR
Ryerson [17] (primary) Ryerson [17] (secondary) Ryerson [17] (tertiary)	TOUCH database-Biogen’s Tysabri Global Safety Database	1988/13,132 3331/15,424 815/23,168	February 2012 to June 2017	Retrospective cohort	Two different groups	PML
Ryerson [32]	TOUCH data (7 US sites)	79/354	Jul-20	Retrospective cohort	Two different groups	MRI activity
Trojano [21]	Belgium, Germany, Spain, France, and Italy	52/54	Dec-10	RCT	Two different groups	Relapses, MRI activity, and confirmed disability worsening
Van Kempen [38]	4 hospitals in Netherlands	51/10	November 2015 to June 2018	Prospective multicenter single arm	Pre vs post switch	MRI activity, relapses and EDSS
Yamout [22]	Lebanon–Iran	85/85	–	Retrospective review study of prospectively followed cohorts	Pre vs post switch	EDSS, ARR, relapses, MRI activity and disability progression

*EID* extended interval dosing, *SID* standard interval dosing, *MS* multiple sclerosis, *PML* progressive multifocal leukoencephalopathy, *EDSS* Expanded Disability Status Scale, *ARR* annualized relapse rate, *NEDA* no evidence of disease activity, *RCT* randomized controlled trial

**Table 2:** Baseline characteristics of the enrolled patients in the included studies

ID	Age, mean (SD), EID/SID	Male/Female, (%/%)		EID/SID durations		Follow-up duration	JCV ( +), *n*		Prior use of DMTs, *n* (%)	
		EID	SID	Interval duration	Mean treatment duration		EID	SID	EID	SID
Bomprezzi [33]	41(10)/41(11)	26/74	28/72	6–8/4 W	Minimum 6 M	6 M	84	150	–	–
Butzkueven [23]	39.9 (9.55)/40.6 (9.4)	32.9/67.1	32.9/67.1	6/4 W	52.32/52.92 M	2 Years	74	68	–	–
Chisari [34]	42.3(13.4)/41.6(11.5)	38.5/61.5	39.9/60.1	39.8/30.8 D	–	12–24 M	31	245	296 (35.3)	326 (26)
De Mercanti [35]	34.4 (9.8)/34.9 (10.7)	–	–	42.2/32.2 D	32/24.64 M	6-12-24 M	66	66	–	–
Foley [4]	40·9 (9·66) /40·3 (9·94)	30/70	27/73	6/4 W	48/48 M	24-48-72 W	52	64	184 (74)	175 (72)
Jeantin [39]	24.93 (7.83)	42.1/57.9		6 W	58.17 M	139.1 M	–	–	–	–
Pelle [40]	39.3 (9.7)/40.2 (10.6)	25/75	21/79	> 6W	–	12 M	19	14	–	–
Riancho [36]	43.41 (10.71)/38.97 (11.1)	13/87		8/4 W	76.68/51.1 M	7 Years	32	–	25(64)	
Ryerson [37]	45.59 (11.67)/45.48 (11.48)	27/73	30/70	7-8W, 5D/4W, 3D-6W, 6D/4W	–	–	563	540	152(17)	1296(12)
Ryerson [17] (primary)	42.9 (11.3)/44.0 (11.0)	31/69	33/67	36.7/30 D	59.9/47.77 M	5 Years	1,988	13٫132	95 (5)	689 (5)
Ryerson [17] (secondary)	43.0 (11.2)/43.9 (11.4)	31/69	34/66	35/29.8 D	53.56/29.02 M		3٫331	15٫424	175 (5)	799 (5)
Ryerson [17] (tertiary)	42.0 (11.4)/43.9 (11.6)	34/66	33/67	43/30.5 D	44/25.04 M		815	23٫168	49 (6)	1,310 (6)
Ryerson [32]	42.24(10.3)/42.48(10.33)	28/72	30/70	6/4 W	–	24 W	–	–	–	–
Trojano [21]	38.7 (8.43)/38.4 (7.84	28.8/71.2	27.8/72.2	12/4 W	3.1/3.2 Years	72 W	–	–	–	–
Van Kempen [38]	40.7 (10.4)/41.6 (10.2)	25/75	40/60	5–7/4 W	4/5 Years	12 M	17	3	–	–
Yamout [22]	33.76 (10.93)	22.4/77.6		≥ 5/4 W	11.83/15.45 M	6–12 M	38		75 (88.2)	

*EID* extended interval dosing, *SID* standard interval dosing, *JCV* John Cunningham virus, *DMT* disease-modifying therapy, *D* days, *M* months, *Y* years

### Quality assessment

All the included studies were of good quality. The details of each domain of the appropriate tool according to study design are provided in Supp. Tables 1, 2, 3. Our GRADE assessment results, detailed in Supp. Table 4, indicated a very low level of certainty of evidence concerning several aspects: specifically, New or newly enlarging T2 hyperintense lesions, Delta EDSS, Patients with clinical relapses in the 12-week subgroup, PML, and Gadolinium-enhancing lesions. Furthermore, the certainty of evidence was deemed low in the case of Patients with clinical relapses in the 5–8-week subgroup. The primary reason for downgrading revolved around imprecision due to wide CIs and the predominance of evidence sourced from observational studies, leading to the overall decrease in confidence in these outcomes. A summary of the major limitations of each study is shown in Supp. Table 5.

### Quantitative results

#### Clinical relapses

**Fig. 2:**
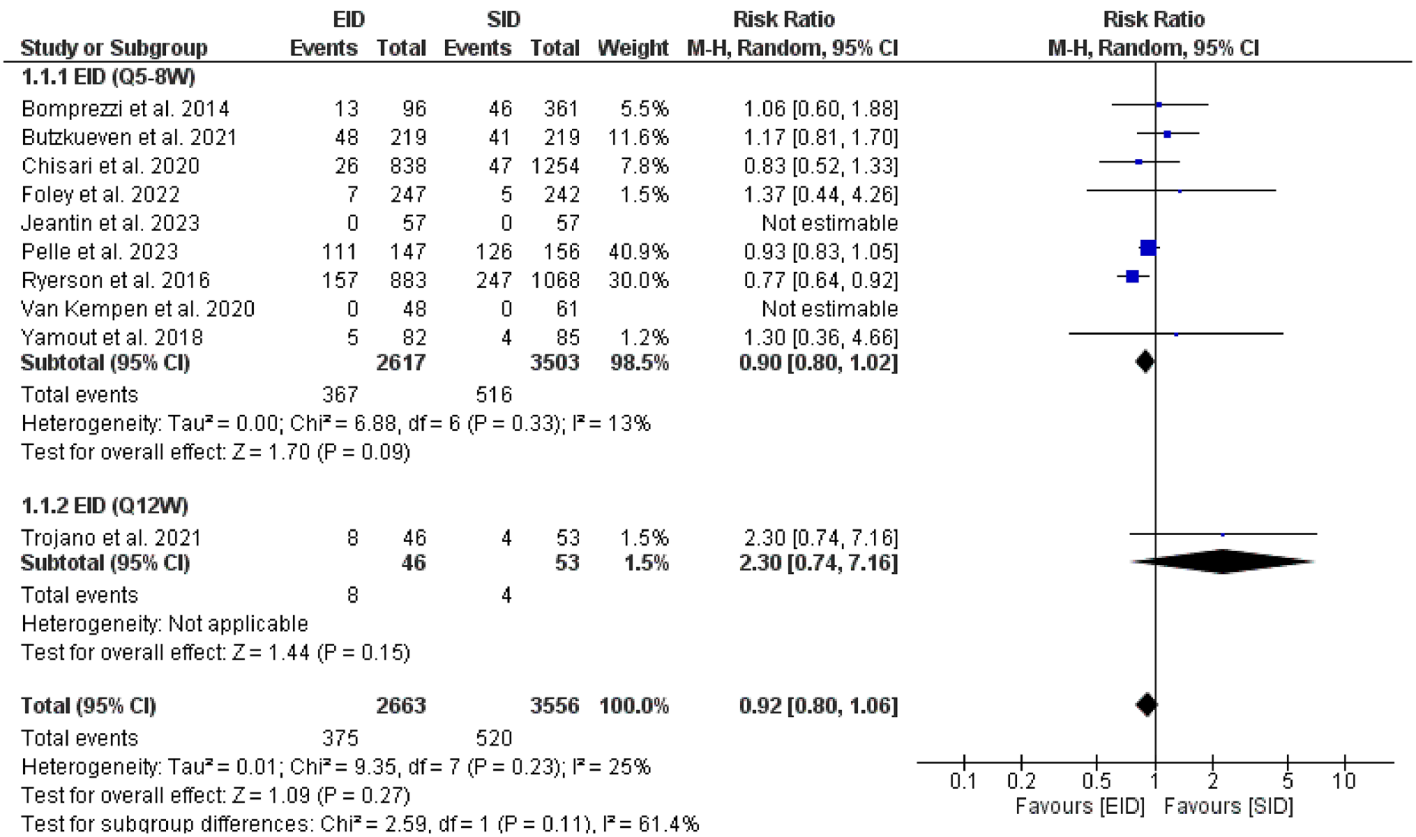
A forest plot of the risk of clinical relapse

The pooled effect of nine studies [22, 23, 33, 34, 37–40] showed no significant difference in the risk of clinical relapses in the EID [Q5-8W] than SID [RR = 0.90, (95%CI 0.80, 1.02), *P* = 0.09]. Heterogeneity was insignificant [*P* = 0.33, I2 = 13%], Fig. 2*.* The funnel plot is provided in the Additional file 1.

#### MRI activity

**Fig. 3:**
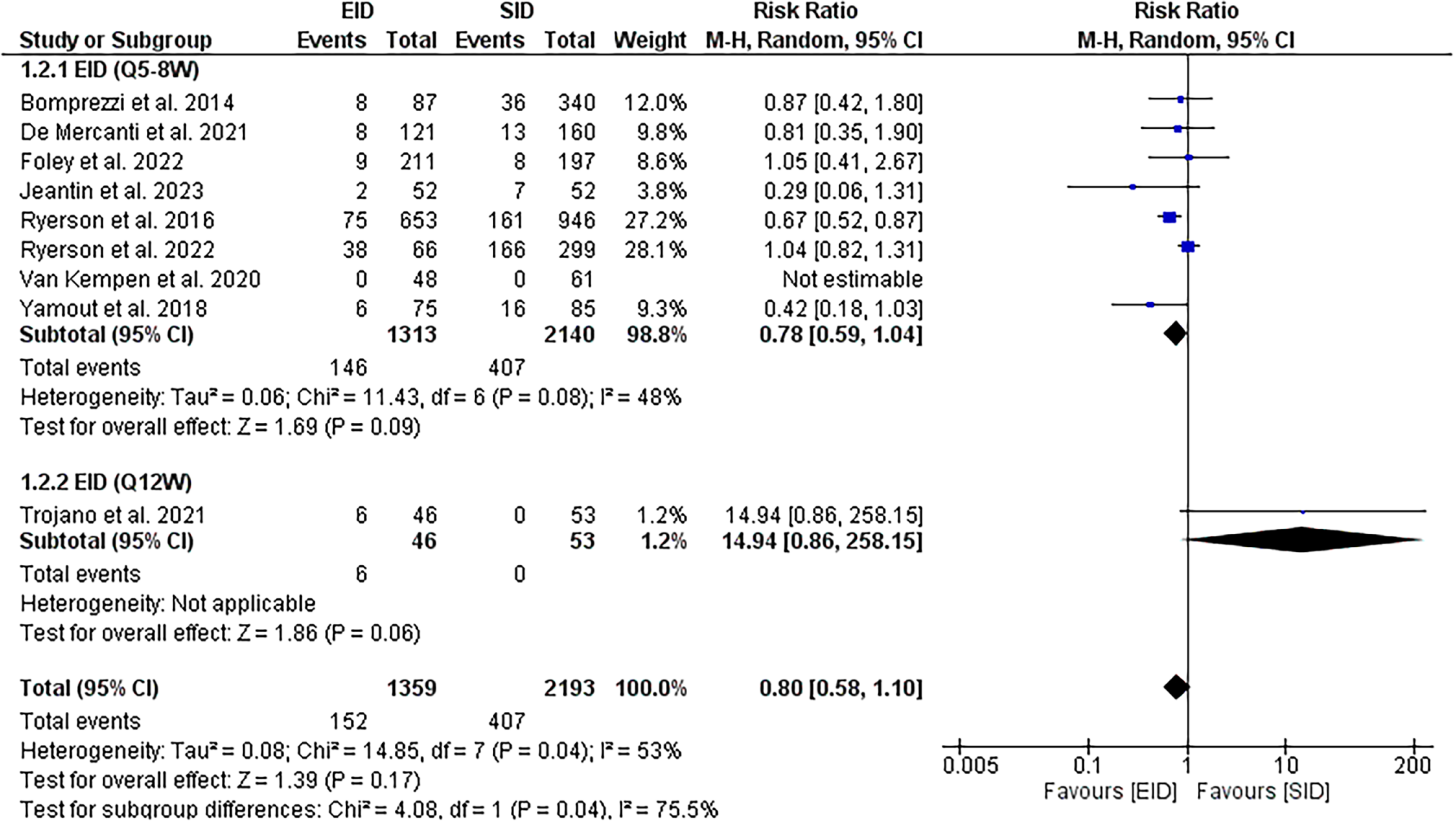
A forest plot of the risk of new or newly enlarging T2 hyperintense lesions

**Fig. 4:**
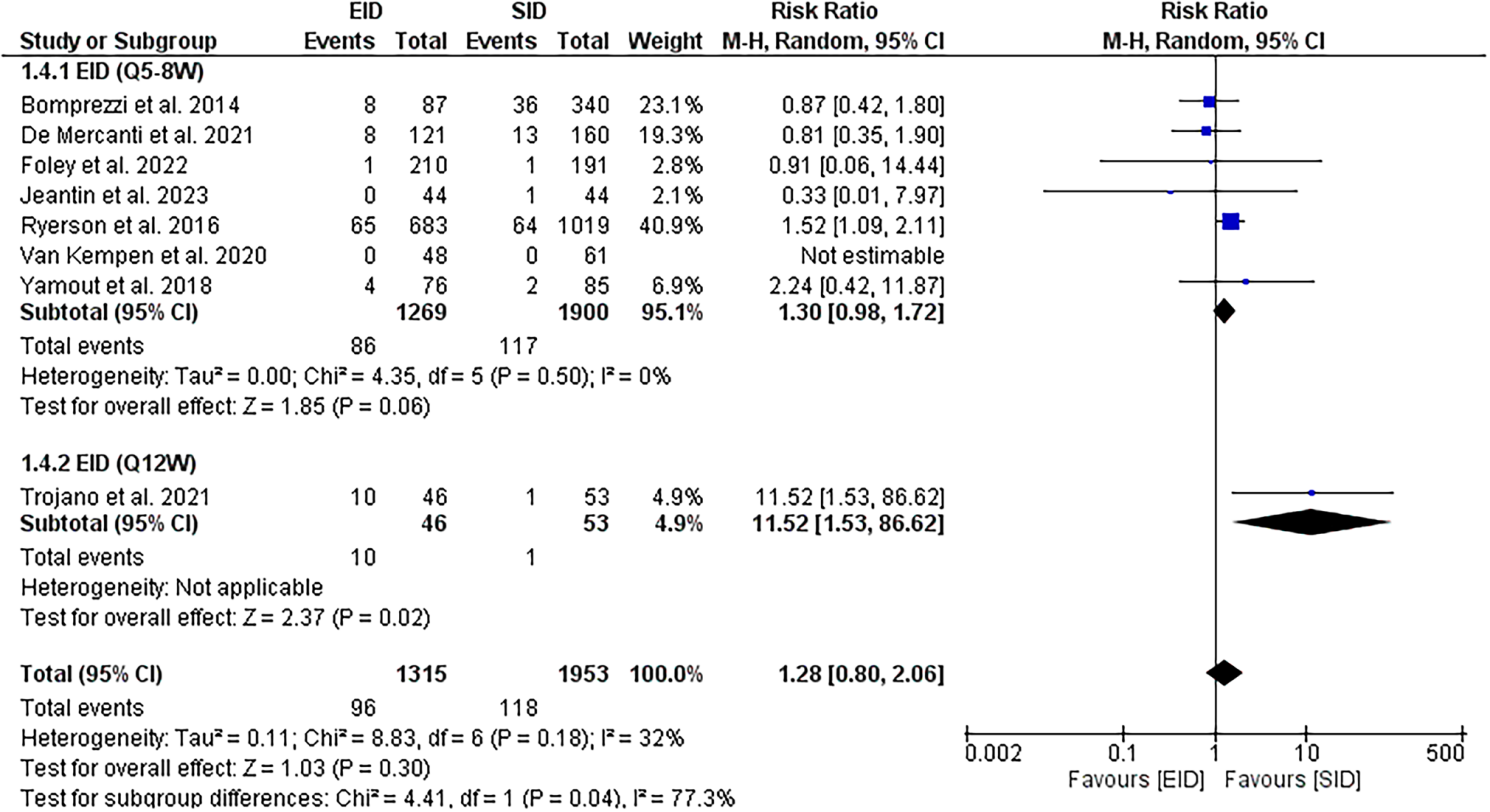
A forest plot of the risk of the gadolinium-enhancing lesion

EID [Q5-8W] showed no significant difference in the risk of new or newly enlarging T2 hyperintense lesions [RR = 0.78, (95%CI 0.59, 1.04), *P* = 0.09]. Insignificant heterogeneity was observed [*P* = 0.08, *I*^2^ = 48%], Fig. 3. The pooled effect showed no significant difference in risk of gadolinium-enhancing lesions between EID [Q5-8W] and SID groups [RR = 1.30, (95%CI 0.98, 1.72), *P* = 0.06] with no heterogeneity [*P* = 0.5, *I*^2^ = 0%], Fig. 4.

#### Expanded disability status scale

**Fig. 5:**
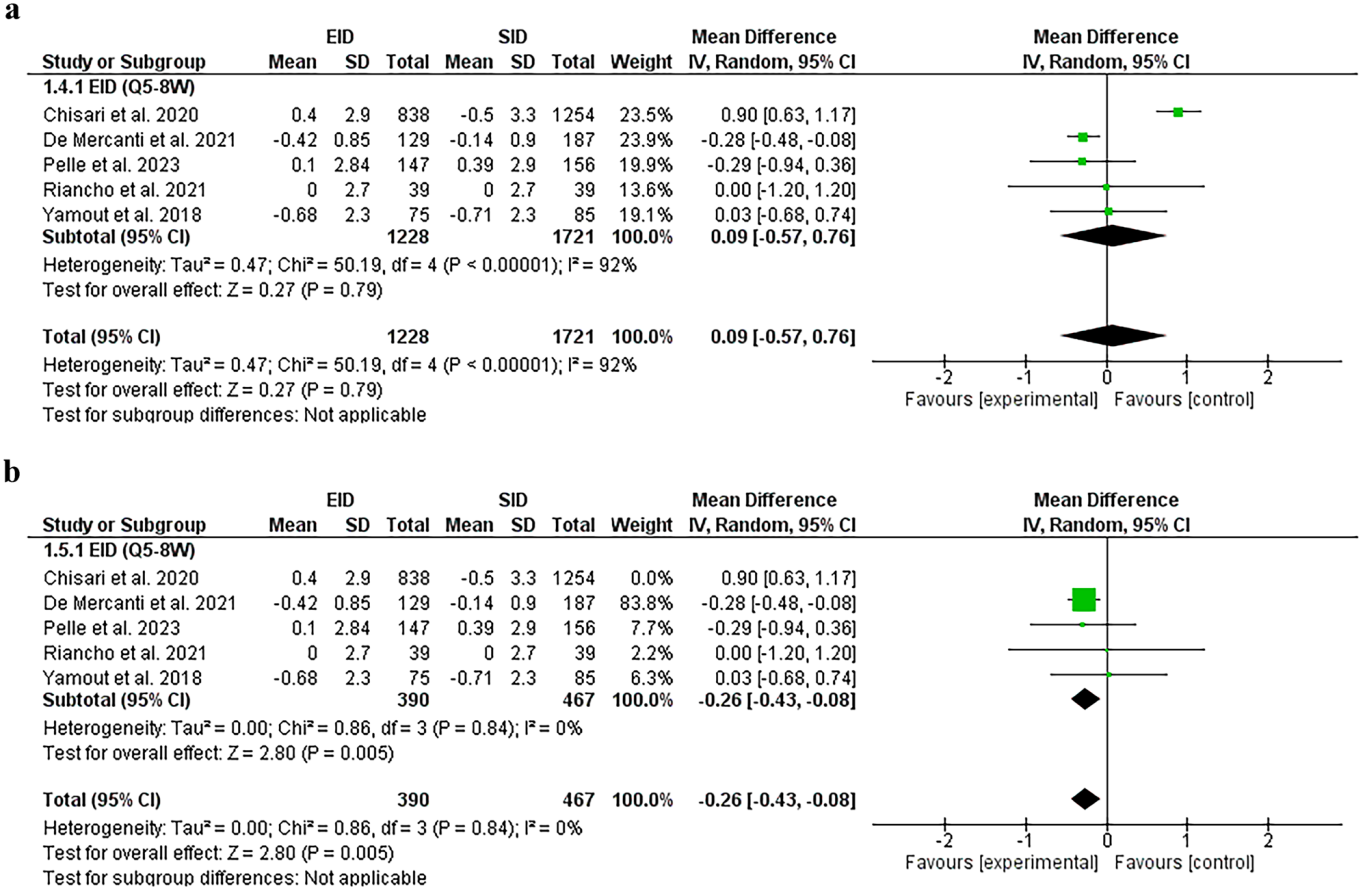
**A** A forest plot of the delta EDSS before sensitivity analysis. **B** A forest plot of the delta EDSS after sensitivity analysis

The delta EDSS was found comparable in both groups [EID [Q5-8W] and SID [MD = 0.09 (95%CI − 0.57, 0.76), *P* = 0.79]. Substantial heterogeneity [*P* < 0.00001, *I*^2^ = 92%] was found and solved by removing Chisari et al. [34] [*P* = 0.84, *I*^2^ = 0%]. After sensitivity analysis, the results favored the EID [Q5-8W] group [MD = − 0.26, (95%CI − 0.43, − 0.08), *P* = 0.005], Fig. 5A, B.

#### Progressive multifocal leukoencephalopathy

**Fig. 6:**
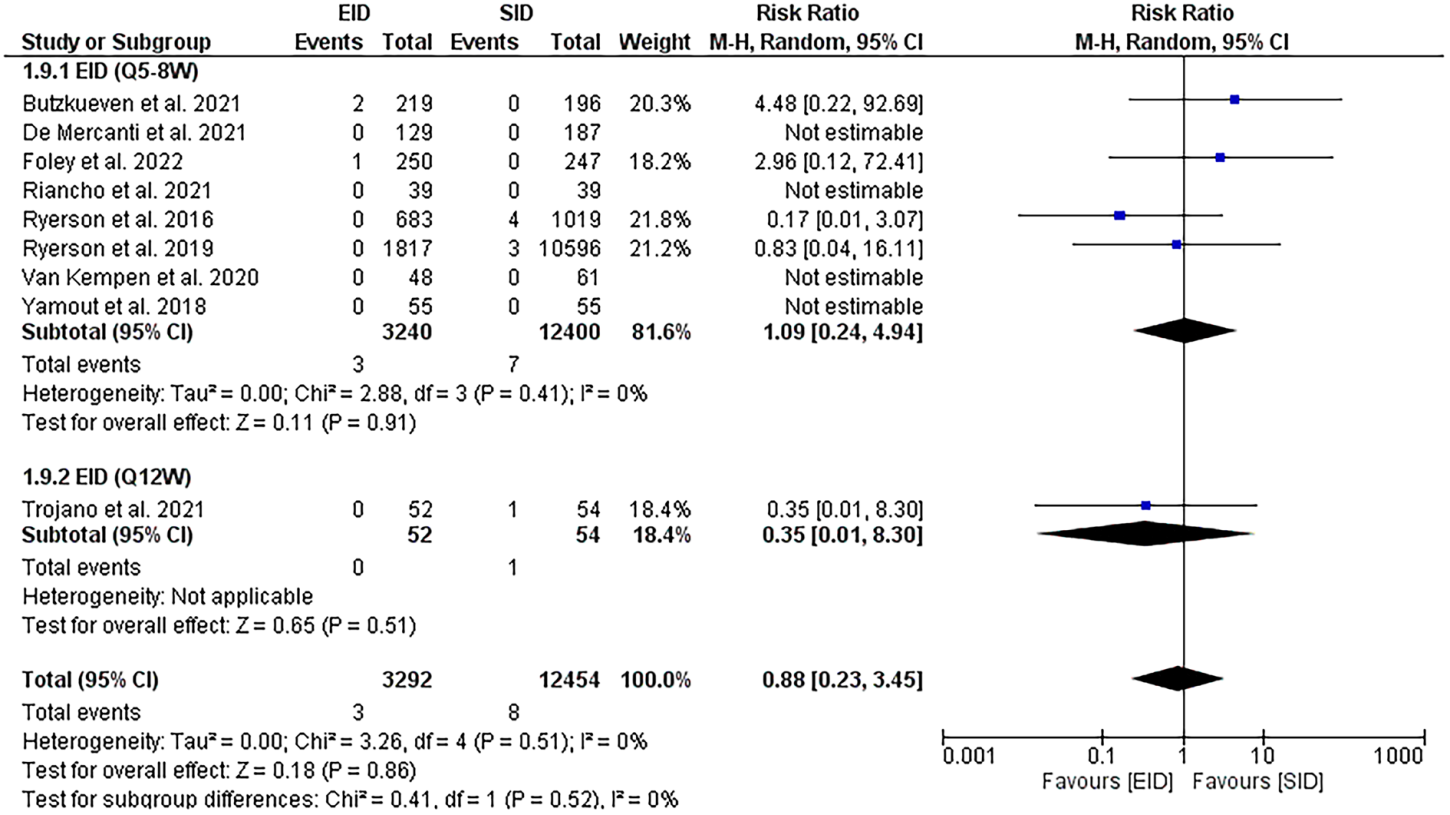
A forest plot of the PML risk

The new PML cases were found to be comparable between both EID [Q5-8W] and SID groups [RR = 1.09, (95%CI 0.24, 4.94], *P* = 0.91) with no heterogeneity [*P* = 0.41, *I*^2^ = 0%], Fig. 6.

## Discussion

EID [Q5-8W] demonstrated non-inferiority in all efficacy outcomes when compared to the standard interval dosing (SID); the risk of clinical relapses, new or newly enlarging T2 hyper-intense lesions, gadolinium-enhancing lesions, EDSS, and lastly, the risk of PML. This study supports the trend toward extending the intervals between infusions of natalizumab while maintaining the drug's effectiveness.

Natalizumab is associated with a decrease in inflammation and improvement in clinical and radiological activity [7]. Although natalizumab is a highly effective drug in MS, it is burdened by the risk of PML [17, 42]. EID has been studied to confirm its superiority in reducing PML. Ryerson et al.—a retrospective cohort with 35,521 JCV + MS patients—provides Class III evidence that longer follow-up [up to ten years] is associated with higher PML risk in natalizumab SID than EID [17]. Some reports showed that this risk is not diminished totally by the EID strategy [43–45]. This is consistent with our meta-analysis finding that showed the PML reduction was statistically insignificant. Thus, patient monitoring should be individualized and tailored separately according to PML risk factors, including previous immunosuppressant use, exposure duration, and anti-JCV antibody index.

EID was associated with reduced nadir serum level of natalizumab, and α4-integrin receptor occupancy, with an increase in α4-integrin cell surface expression. That may explain the protective effect of EID against JCV reactivation; the free α4-integrin receptors may enhance the immunosurveillance of JCV and prevent PML [20]. The serum concentration of 2 μg/mL has been considered adequate to maintain efficacy in most MS patients with a receptor saturation range of 70–100% [14]. Ryerson et al. found that EID maintained receptor saturation within the therapeutic range for most patients. They found a tendency of suboptimal trough saturation in patients on EID with high body mass index. So they need closer clinical and MRI follow-ups [19]. Johnsson et al. measured the change in the serum neurofilament light [sNfL], and they concluded that EID did not increase the axonal damage [46].

Butzkueven et al. found comparable real-world efficacy in MS patients with EID after ≥ 1 year of SID [23]. Several trials found similar results [4, 34, 37, 38]. Ryerson et al. found the same results on the quantitative MRI metrics [32]. However, the most recent RCT revealed a numerical variance at week 72 in the estimated T2 hyperintense lesions between the EID and SID groups [4]. These differences are not clinically meaningful as ultimate T2 lesion numbers influenced the two cases, and a disproportion in rescue therapy [optional] recipients influenced the cases. Bomprezzi et al. showed comparable relapse rates between the two groups [33]. Long-term therapy with EID of natalizumab in Riancho et al. preserved efficacy and safety for over 7 years [36]. On the other hand, Trojano et al. found that an extended interval dosing (EID) regimen of 12 weeks, whether administered intravenously or subcutaneously, was associated with increased MRI disease activity and a greater number of clinical relapses [21]. Their results were in line with other studies suggesting that natalizumab loses its efficacy with reactivation of the disease after approximately 8–12 weeks [16]. The interpretation of this aspect remains open to debate and warrants additional investigation for a more comprehensive understanding of the dynamics between natalizumab dosing intervals and the preservation of therapeutic efficacy.

From the economic point of view, natalizumab EID is associated with lower costs; directly by decreasing the number of infusions per year, decreasing PML and disability-related costs, and decreasing outpatient visits. It also indirectly decreases the social costs and the burden of patient sick leave and caregiver costs [47].

To our knowledge, this is the first meta-analysis pooling the current evidence on this point. However, the included studies exhibit several common limitations. First, most of the included studies are retrospective, which introduces inherent biases and may limit the ability to control for confounding variables. Second, small sample sizes across multiple studies are acknowledged, impacting the robustness of safety outcome comparisons and statistical power, especially for rare events such as progressive multifocal leukoencephalopathy (PML). Third, several studies note potential biases related to the non-randomized design, with concerns about patient selection favoring those with less active disease transitioning to extended interval dosing (EID). Fourth, some studies exclude or lack standardized evaluations for certain parameters, such as MRI data. Lastly, variations in dosing intervals, criteria for MRI surveillance, and heterogeneity in patient characteristics contribute to the overall complexity and potential confounders in the interpretation of results across the studies. We tried to overcome this variability by dividing the EID group into two subgroups [5–8 weeks–12 weeks], extracting the data corresponding to the same follow-up periods as much as possible.

There is a possible overlap between Ryerson (2022) and Ryerson (2016), however, no clear identification of overlapping data registers was addressed [32, 37]. A Summary of each study limitations are summarised in Additional file 1: Table S5.

Based on the current evidence, natalizumab effectiveness is preserved under the EID regimen [up to eight weeks] in terms of comparable risks of clinical relapses, MRI lesions, EDSS and PML. However, it is crucial to approach these findings with caution, given the inherent limitations of the included studies, such as small sample sizes and the predominantly retrospective design. The observed heterogeneity across these studies introduces a level of uncertainty that warrants careful consideration. Furthermore, well-conducted high-quality prospective studies with extended follow-up periods are still warranted, particularly for a more comprehensive assessment of PML risk. Additionally, exploring the differential efficacy of various dosing intervals of natalizumab separately through rigorous RCTs will provide a more nuanced understanding of the optimal treatment strategy.

### Supplementary Information

Below is the link to the electronic supplementary material.

Supplementary file1 (DOCX 595 KB)

## Data Availability

The datasets used and/or analyzed during the current study are available from the corresponding author upon reasonable request.
