# Supplementary material for: Safety and efficacy of extended versus standard interval dosing of natalizumab in multiple sclerosis patients: a systematic review and meta-analysis
Source: Acta Neurol Belg. 2024 Mar 8;124(2):407–17. doi: 10.1007/s13760-024-02480-6 (PMC10965735; doi:10.1007/s13760-024-02480-6)
Supplement: Supplementary file 1 — Supplementary file1 (DOCX 595 KB) [file 13760_2024_2480_MOESM1_ESM.docx]

**Table S1.** Quality assessment of the observational studies.

| ID | **New-Castle Ottawa Scale (NOS) for cohort studies** | | | | | | | | |
| --- | --- | --- | --- | --- | --- | --- | --- | --- | --- |
|  | Selection | | | | Comparability | Outcome | | | |
|  | Representativeness | Selection of the non-exposed | Ascertainment | The outcome does not present at start |  | Assessment | follow-up long | Adequacy of follow up | Quality score |
| Bomprezzi 2014 | * | * | * | - | ** | * | * | * | Good quality |
| Butzkueven 2021 | * | * | * | - | ** | * | * | * | Good quality |
| Chisari 2020 | * | * | * | - | * | * | * | * | Good quality |
| De Mercanti 2021 | * | * | * | - | * | * | * | * | Good quality |
| Jeantin 2023 | - | * | * | - | ** | * | * | * | Good quality |
| Pelle 2023 | * | * | * | - | ** | * | * | * | Good quality |
| Riancho 2021 | * | * | * | - | * | * | * | * | Good quality |
| Ryerson 2016 | * | * | * | - | ** | * | - | * | Good quality |
| Ryerson 2019 | * | * | * | - | * | * | * | * | Good quality |
| Ryerson 2022 | * | * | * | - | ** | * | * | * | Good quality |
| Yamout 2018 | * | * | * | - | ** | * | * | * | Good quality |
|  |  |  |  |  |  |  |  |  |  |

**Table S2.** Quality assessment of the single-arm trial.

| ID | **NIH Quality Assessment Tool for Single arm studies** | | | | | | | | | | | | | |
| --- | --- | --- | --- | --- | --- | --- | --- | --- | --- | --- | --- | --- | --- | --- |
|  | 1 | 2 | 3 | 4 | 5 | 6 | 7 | 8 | 9 | 10 | 11 | 12 | Total scores | Quality |
| Van kempen 2020 | Yes | Yes | No | Yes | Yes | Yes | Yes | No | Yes | Yes | Yes | NA | 10 | Good quality |

1. Was the research question or objective in this paper clearly stated?

2. Were eligibility/selection criteria for the study population prespecified and clearly described?

3. Were the participants in the study representative of those who would be eligible for the test/service/intervention in the general or clinical population of interest?

4. Were all eligible participants that met the prespecified entry criteria enrolled?

5. Was the sample size sufficiently large to provide confidence in the findings?

6. Was the test/service/intervention clearly described and delivered consistently across the study population?

7. Were the outcome measures prespecified, clearly defined, valid, reliable, and assessed consistently across all study participants?

8. Were the people assessing the outcomes blinded to the participants' exposures/interventions?

9. Was the loss to follow-up after baseline 20% or less? Were those lost to follow-up accounted for in the analysis?

10. Did the statistical methods examine changes in outcome measures from before to after the intervention? Were statistical tests done that provided p values for the pre-to-post changes?

11. Were outcome measures of interest taken multiple times before the intervention and multiple times after the intervention (i.e., did they use an interrupted time-series design)?

12. If the intervention was conducted at a group level (e.g., a whole hospital, a community, etc.) did the statistical analysis take into account the use of individual-level data to determine effects at the group level?

Total score: Yes = 1 // No = 0.5 // NR & NA & CD = 0

Quality rating: good (9.5-12 point) or fair (6.5-9 point) or poor (6-0 points).

**Table S3.** Quality assessment of the randomized clinical trials.

| ID | The Cochrane Collaboration’s tool for assessing the risk of bias | | | | | | |
| --- | --- | --- | --- | --- | --- | --- | --- |
|  | Random sequence generation (selection bias) | Allocation concealment (selection bias) | Blinding of participants and personnel (performance bias) | Blinding of outcome assessment (Detection bias) | Incomplete outcome data (attrition bias) | Selective reporting (reporting bias) | Other Bias |
| Foley 2022 | Low risk | High risk | High risk | Low risk | Low risk | Low risk | Unclear risk |
| Trojano 2021 | Unclear risk | Unclear risk | Low risk | Low risk | Low risk | Low risk | Unclear risk |

**Table S4.** GRADE assessment for the quality of the evidence resulted from the meta-analysis.

| **Certainty assessment** | | | | | | | **№ of patients** | | **Effect** | | **Certainty** | **Importance** |
| --- | --- | --- | --- | --- | --- | --- | --- | --- | --- | --- | --- | --- |
| **№ of studies** | **Study design** | **Risk of bias** | **Inconsistency** | **Indirectness** | **Imprecision** | **Other considerations** | **EID** | **SID** | **Relative (95% CI)** | **Absolute (95% CI)** |  |  |
| **New or newly enlarging T2 hyperintense lesions (total analysis)** | | | | | | | | | | | | |
| 9 | observational studies | not serious | serious^a^ | not serious | serious^b^ | none | 152/1359 (11.2%) | 407/2193 (18.6%) | **RR 0.80** (0.58 to 1.10) | **37 fewer per 1,000** (from 78 fewer to 19 more) | ⨁◯◯◯ Very low | CRITICAL |
| **New or newly enlarging T2 hyperintense lesions (EID (Q5-8W))** | | | | | | | | | | | | |
| 8 | observational studies | not serious | serious^a^ | not serious | not serious | none | 146/1313(11.1%) | 407/2140(19.0%) | **RR 0.78** (0.59 to 1.04) | **42 fewer per 1,000** (from 78 fewer to 8 more) | ⨁◯◯◯ Very low | CRITICAL |
| **New or newly enlarging T2 hyperintense lesions (homogenous, total analysis)** | | | | | | | | | | | | |
| 1 | RCT | not serious | not serious | not serious | very serious^b^ | none | 6/46 (13.0%) | 0/53 (0.0%) | **RR 14.94 (0.86 to 258.15)** | **0 fewer per 1,000** (from 0 fewer to 0 fewer) | ⨁◯◯◯ Very low | CRITICAL |
| **Delta EDSS (heterogenous)** | | | | | | | | | | | | |
| 5 | observational studies | not serious | very serious^c^ | not serious | not serious | none | 1228 | 1721 | - | MD **0.09 higher** (0.57 lower to 0.76 higher) | ⨁◯◯◯ Very low | IMPORTANT |
| **Delta EDSS (homogenous)** | | | | | | | | | | | | |
| 4 | observational studies | not serious | not serious | not serious | not serious | none | 390 | 467 | - | MD **0.26 lower** (0.43 lower to 0.08 lower) | ⨁⨁◯◯ Low | IMPORTANT |
| **Patients with clinical relapses (total analysis)** | | | | | | | | | | | | |
| 10 | observational studies | not serious | not serious | not serious | serious^b^ | none | 375/2663 (14.08%) | 520/3556 (14.6%) | **RR 0.92** (0.80 to 1.06) | **12 fewer per 1,000** (from 29 fewer to 9 or more) | ⨁◯◯◯ Very low | CRITICAL |
| **Patients with clinical relapses - (EID (Q5-8W) subgroup)** | | | | | | | | | | | | |
| 9 | observational studies | not serious | not serious | not serious | not serious | none | 367/2617 (14.02%) | 516/3503 (14.7%) | **RR 0.90** (0.80 to 1.02) | **15 fewer per 1,000** (from 29 fewer to 3 more) | ⨁⨁◯◯ Low | CRITICAL |
| **Patients with clinical relapses - (EID (12 W) subgroup)** | | | | | | | | | | | | |
| 1 | RCT | not serious | not serious | not serious | very serious^b^ | none | 8/46 (17.4%) | 4/53 (7.5%) | **RR 2.30 (0.74 to 7.16)** | **98 fewer per 1,000** (from 20 fewer to 465 more) | ⨁◯◯◯ Very low | CRITICAL |
| **PML (total analysis)** | | | | | | | | | | | | |
| 9 | observational studies | not serious | not serious | not serious | very serious^b^ | none | 3/3292(0.1%) | 8/12454 (0.1%) | **RR 0.88** (0.23 to 3.45) | **0 fewer per 1,000** (from 0 fewer to 2 more) | ⨁◯◯◯ Very low | CRITICAL |
| **PML - (EID (Q5-8W) subgroup)** | | | | | | | | | | | | |
| 8 | observational studies | not serious | not serious | not serious | very serious^b^ | strong association | 3/3240 (0.1%) | 7/12400 (0.1%) | **RR 1.09** (0.24 to 4.94) | **0 fewer per 1,000** (from 0 fewer to 2 more) | ⨁◯◯◯ Very low | CRITICAL |
| **PML - (EID (12 W) subgroup)** | | | | | | | | | | | | |
| 1 | RCT | not serious | not serious | not serious | very serious^b^ | none | 0/52 (0.0%) | 1/54 (1.9%) | **RR 0.35 (0.01 to 8.30)** | **12 fewer per 1,000** (from 18 fewer to 135 more) | ⨁◯◯◯ Very low | CRITICAL |
| **Gadolinium-enhancing lesions (total analysis)** | | | | | | | | | | | | |
| 8 | observational studies | not serious | very serious^c^ | not serious | very serious^b^ | none | 96/1315 (7.3%) | 118/1953 (6.0%) | **RR 1.28** (0.08 to 2.06) | **17 more per 1,000** (from 12 fewer to 64 more) | ⨁◯◯◯ Very low | CRITICAL |
| **Gadolinium-enhancing lesions - (EID (Q5-8W) subgroup)** | | | | | | | | | | | | |
| 7 | observational studies | not serious | very serious^c^ | not serious | very serious^b^ | none | 86/1296 (6.6%) | 117/1900 (6.2%) | **RR 1.30** (0.98 to 1.72) | **18 fewer per 1,000** (from 1 fewer to 44 more) | ⨁◯◯◯ Very low | CRITICAL |
| **Gadolinium-enhancing lesions (EID (Q 12W) subgroup)** | | | | | | | | | | | | |
| 1 | RCT | not serious | not serious | not serious | very serious^b^ | none | 10/46 (21.7%) | 1/53 (1.9%) | **RR 11.52 (1.53 to 86.62)** | **198 fewer per 1,000** (from 10 more to 1000 more) | ⨁◯◯◯ Very low | CRITICAL |

**CI:** confidence interval; **MD:** mean difference; **RR:** risk ratio

#### Explanations

a. High level of heterogeneity (between 50-70%)

b. Wide confidence interval

c. Extremely high level of heterogeneity (more than 70%)

**Table S5.** Limitations of included studies.

| **ID** | **Major limitations** |
| --- | --- |
| Bomprezzi 2014 | - Retrospective Nature. - The non-randomized nature of the study, with a potential predisposition to skewed results as patients with less active disease may be more likely to switch to EID. - Small sample size. |
| Butzkueven 2021 | - Retrospective Nature. - Small sample sizes in the study are acknowledged as insufficient for robust safety outcome comparisons, especially for rare events. - Both PML cases in this study had known risk factors, including anti-JCV antibodies, natalizumab exposure beyond 2 years, and prior use of immunosuppressants, placing them in the highest PML risk category. |
| Chisari 2020 | - Retrospective Nature. - Patients with less aggressive MS may be more likely to transition to the EID schedule, indicated by longer disease duration and higher NTZ administrations in EID compared to SID. - Exclusion of MRI data due to susceptibility to variability in evaluators and MRI machines. - The use of apparently early EID (≥33 days) might impact the estimation of wider interval dosing schedules on NTZ effectiveness. |
| De Mercanti 2021 | - Retrospective Nature. - Heterogeneity in dosing intervals (wider in EID vs SID cohorts) and non-uniform criteria for MRI surveillance further complicate the analysis. - Absence of a standardized scanning protocol or central reading adds to the complexity. - The basis for recommending standard natalizumab dosing vs extended interval dosing varies, adding considerable confounders. |
| Foley 2022 | - Sample size calculations and inferences were based on the assumption of a true mean number of new T2 hyperintense lesions in the 4-week dosing group as 0·3 from previous trials. - Insufficient size and duration of the trial to provide informative data on rare adverse events, such as progressive multifocal leukoencephalopathy. |
| Jeantin 2023 | - Retrospective Nature. - The design compared the same patients before and after the extension of natalizumab interval dosing over a brief period. Although certain patient characteristics were similar, it may not capture long-term effects. - Small sample size. |
| Pelle 2023 | - Retrospective Nature. - Potential bias in treatment decisions based on factors like MS severity and PML risk, influencing the choice between EID and SID. - Possible center effects due to the absence of a standardized evaluation protocol, leading to variations in EDSS scoring and MRI evaluations. - Small sample size and failure to reach the planned SID to EID ratio. - Fewer than expected relapses, impacting statistical power needed for noninferiority demonstration. |
| Riancho 2021 | - Retrospective Nature. - Lack of a comparison control group and reliance on observational data. - Limited by a small sample size, impeding further subgroup analyses. |
| Ryerson 2016 | - Retrospective Nature. - the inability to control for baseline disease characteristics and - possible selection bias, with likely less aggressive patients with MS moved on to the EID schedule. - EDSS was not utilized for disability progression assessment due to inconsistency across the nine MS centers. |
| Ryerson 2019 (primary) Ryerson 2019 (secondary) Ryerson 2019 (tertiary) | - Retrospective Nature. - Limitations arise from patients on EID receiving more natalizumab doses than those on SID, potentially introducing a selection bias favoring more PML cases in the EID cohorts. - Patients with >2 years of SID treatment may inherently have a reduced risk of PML, creating a bias favoring fewer PML cases in the EID groups. - Lack of anti-JCV antibody index data for all TOUCH patients. |
| Ryerson 2022 | - Retrospective Nature. - Clinical disease covariates, such as relapses or disability progression assessments, were not available, leading to potential bias from unmeasured factors. - Reasons for patients switching to natalizumab EID or remaining on SID are unknown, potentially reflecting differences in disease activity between the groups. |
| Trojano 2021 | - No formal statistical comparisons were prespecified. - Patients were not natalizumab naive before study enrollment. |
| Van Kempen 2020 | - Exclusively included patients with stable disease in the year before the study which limits the generalizability of results to a broader patient population. |
| Yamout 2018 | - Retrospective Nature - Sample size and treatment duration limitations preclude assessing PML risk in either group. |

Abbreviations: EID, extended interval dosing; SID, standard interval dosing; MS, multiple sclerosis; PML, progressive multifocal leukoencephalopathy; EDSS, Expanded Disability Status Scale; NTZ, natalizumab.


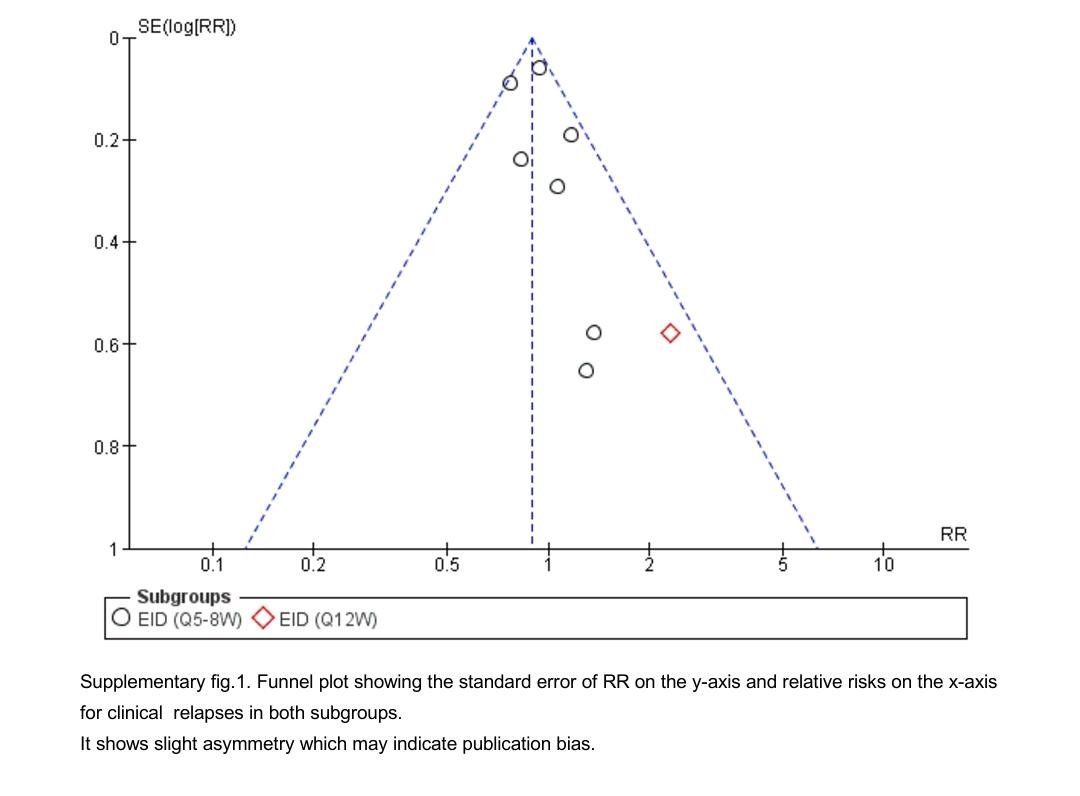


Supplementary fig.1. Funnel plot showing the standard error of RR on the y-axis and relative risks on the x-axis for clinical relapses in both subgroups.

It shows slight asymmetry which may indicate publication bias.
